# P-1434. Geostatistical Analysis of Rocky Mountain Spotted Fever Outbreak in Mexican Pediatric Patients: A Retrospective Observational Study

**DOI:** 10.1093/ofid/ofae631.1608

**Published:** 2025-01-29

**Authors:** Lindsay A Concha-Mora, Jose Eduardo Mares-Gil, Oscar Octavio Loya-Guerrero, Cesar Antonio Ramos-Ortiz, Pablo D Treviño-Valdez, Oscar Tamez-Rivera

**Affiliations:** Pediatric Residency Program, Programa Multicéntrico de Especialidades Médicas ITESM- SSNL, Tecnológico de Monterrey. Escuela de Medicina y Ciencias de la Salud . Monterrey, México, Monterrey, Nuevo Leon, Mexico; Pediatric Residency Program, Programa Multicéntrico de Especialidades Médicas ITESM- SSNL, Tecnológico de Monterrey. Escuela de Medicina y Ciencias de la Salud . Monterrey, México, Monterrey, Nuevo Leon, Mexico; Tec de Monterrey, Monterrey, Nuevo Leon, Mexico; Secretaría de Salud Nuevo León / Instituto Tecnológico y de Estudios Superiores de Monterrey, Monterrey, Nuevo Leon, Mexico; Tecnologico de Monterrey, Escuela de Medicina y Ciencias de la Salud, Monterrey, Nuevo Leon, Mexico; Tecnologico de Monterrey, Escuela de Medicina y Ciencias de la Salud, Monterrey, Nuevo Leon, Mexico

## Abstract

**Background:**

Rocky Mountain Spotted Fever (RMSF) is a bacterial vector-borne disease transmitted through infected ticks. Northern Mexico is currently experiencing an alarming rise of RMSF cases associated with high mortality, affecting the pediatric population. Geographic Information Systems (GIS) characterize geotemporal trends of infectious diseases and offer valuable information for public health strategies. Accurately pinpointing high burden areas of RMSF and tracking its geographical behavior throughout time was crucial to mitigate the outbreak.

Heat map of pediatric RMSF cases from August 2022 and September 2023 at the Pediatric Reference of Hospital of Nuevo León, Mexico

**Figure:**
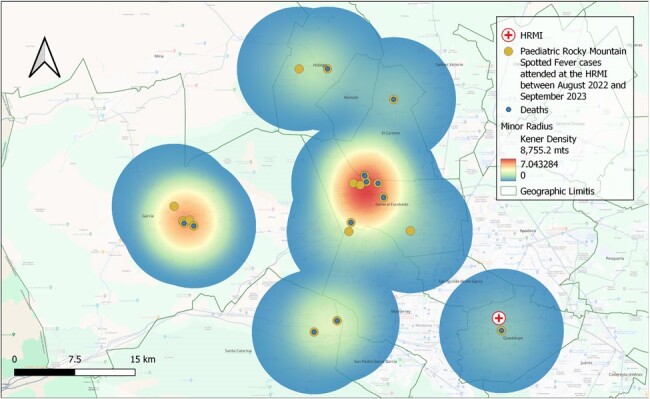

Heat map was projected with the minor ratio obtained by Kernel distribution

**Methods:**

Geographical and clinical data of patients < 16 yr with confirmed RMSF diagnosed at the Pediatric reference hospital (HRMI) in NL, Mx from Aug 2022 to Sep 2023 were included. HRMI admitted the majority of affected children during the RMSF outbreak. QGIS® was used to map the geographical distribution of cases. The nearest neighbor index (NNI) was calculated, as well as the Kernel density estimation (KDE) to assess the influence ratio. Regional marginalization indices were obtained from official authorities. Results were visualized with heat maps.

Map of Distance of pediatric RMSF cases from August 2022 and September 2023 to the Pediatric Reference of Hospital (HRMI), Nuevo León, Mexico

**Figure:**
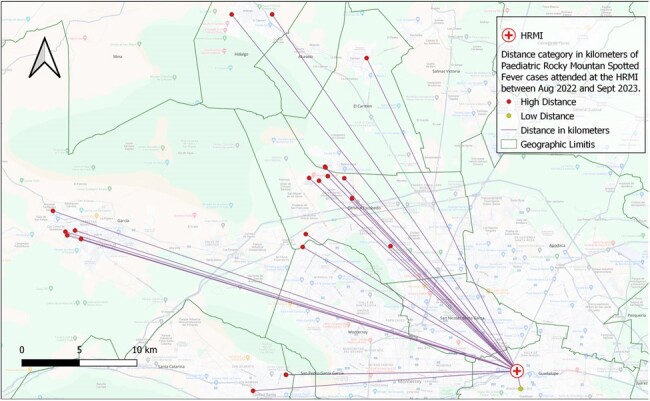

Distance was projected in kilometers, the mean distance case-to-hospital was 24.1 km with a SD ± 10.1km, the lowest in 1.5 km and the highest in 42.6 km

**Results:**

Total of 23 subjects were included. Mean age: 8 years (± 3.3). Most (82%) had a positive history of contact with ticks. Time from symptom onset 4.9 ± 1.8 days. High mortality (65.2%) despite in-hospital treatment. NNI: 0.54 (z-score -4.1), showing a statistically significant cluster distribution (p < 0.05). Mapping was performed with a KDE ratio of 8755 m, demonstrating two main geographical hotspots of Ped-RMSF. Case-to-hospital distance analysis shows a mean of 24.1 km (± 10.1km). Most cases located in areas with medium (65%) and high (30%) marginalization indices. Majority (95%) of cases lived >10 km from HRMI, where the diagnosis was made.

Map of Correlation of RMSF cases from August 2022 and September 2023 at the Pediatric Reference of Hospital of Nuevo León, Mexico and de Margination Index

**Figure:**
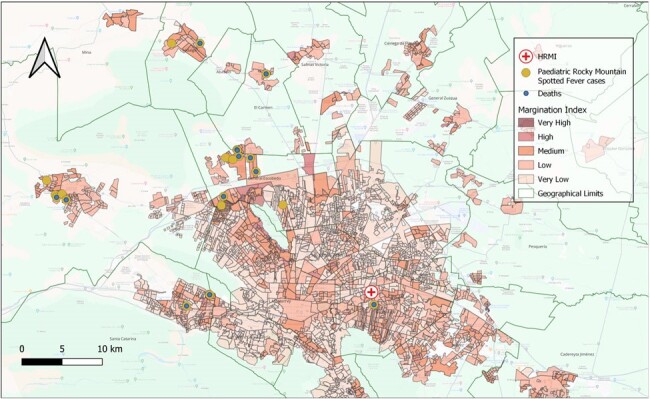

Margination Index was obtain from CONAPO official data (2022). Most cases were located in areas with medium (65%) and high (30%) marginalization indices

**Conclusion:**

The dynamism of vector-borne diseases requires advanced epidemiological surveillance. Through GIS-based analyses we were able to locate high burden areas of Ped-RMSF sharing them with local health authorities for the implementation of vector control strategies. The clustering pattern demonstrated a non-random distribution of cases, highlighting the importance of known factors involved in the transmission of diseases, like marginalization indices. Our study highlights the relevance of GIS use in outbreak response strategies.

**Figure:**
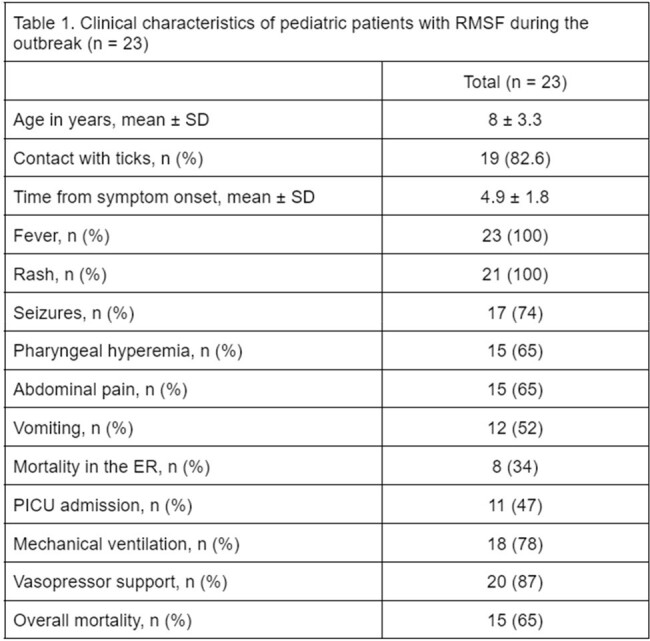

Clinical, managment, outcomes and mortality of Pediatric RMSF Outbreak cases from August 2022 and September 2023 at the Pediatric Reference of Hospital of Nuevo León, Mexico

**Disclosures:**

**All Authors**: No reported disclosures

